# Clinical M2 Macrophage-Related Genes Can Serve as a Reliable Predictor of Lung Adenocarcinoma

**DOI:** 10.3389/fonc.2022.919899

**Published:** 2022-07-22

**Authors:** Chaojie Xu, Lishan Song, Yubin Yang, Yi Liu, Dongchen Pei, Jiabang Liu, Jianhua Guo, Nan Liu, Xiaoyong Li, Yuchen Liu, Xuesong Li, Lin Yao, Zhengjun Kang

**Affiliations:** ^1^ The Fifth Affiliated Hospital of Zhengzhou University, Zhengzhou University, Zhengzhou, China; ^2^ Peking University First Hospital, Peking University, Beijing, China; ^3^ Shenzhen Institute of Translational Medicine, Health Science Center, The First Affiliated Hospital of Shenzhen University, Shenzhen Second People’s Hospital, Shenzhen, China; ^4^ Shenzhen Institute of Translational Medicine, Shenzhen Second People’s Hospital, The First Affiliated Hospital of Shenzhen University, Shenzhen, China; ^5^ College of Pharmacy, Shantou University School of Medicine, Shantou, China

**Keywords:** M2 macrophages, lung adenocarcinoma, WGCNA, risk score, immunotherapy

## Abstract

**Background:**

Numerous studies have found that infiltrating M2 macrophages play an important role in the tumor progression of lung adenocarcinoma (LUAD). However, the roles of M2 macrophage infiltration and M2 macrophage-related genes in immunotherapy and clinical outcomes remain obscure.

**Methods:**

Sample information was extracted from TCGA and GEO databases. The TIME landscape was revealed using the CIBERSORT algorithm. Weighted gene co-expression network analysis (WGCNA) was used to find M2 macrophage-related gene modules. Through univariate Cox regression, lasso regression analysis, and multivariate Cox regression, the genes strongly associated with the prognosis of LUAD were screened out. Risk score (RS) was calculated, and all samples were divided into high-risk group (HRG) and low-risk group (LRG) according to the median RS. External validation of RS was performed using GSE68571 data information. Prognostic nomogram based on risk signatures and other clinical information were constructed and validated with calibration curves. Potential associations of tumor mutational burden (TMB) and risk signatures were analyzed. Finally, the potential association of risk signatures with chemotherapy efficacy was investigated using the pRRophetic algorithm.

**Results:**

Based on 504 samples extracted from TCGA database, 183 core genes were identified using WGCNA. Through a series of screening, two M2 macrophage-related genes (*GRIA1* and *CLEC3B*) strongly correlated with LUAD prognosis were finally selected. RS was calculated, and prognostic risk nomogram including gender, age, T, N, M stage, clinical stage, and RS were constructed. The calibration curve shows that our constructed model has good performance. HRG patients were suitable for new ICI immunotherapy, while LRG was more suitable for CTLA4-immunosuppressive therapy alone. The half-maximal inhibitory concentrations (IC50) of the four chemotherapeutic drugs (metformin, cisplatin, paclitaxel, and gemcitabine) showed significant differences in HRG/LRG.

**Conclusions:**

In conclusion, a comprehensive analysis of the role of M2 macrophages in tumor progression will help predict prognosis and facilitate the advancement of therapeutic techniques.

## Introduction

As one of the cancers with the highest incidence in the world, lung cancer has seriously threatened human life and health (1). According to the National Cancer Institute (NCI), lung cancer is already the most common cause of death among all cancers, with about 350 people dying from lung cancer every day in 2022 (2). Lung adenocarcinoma (LUAD), the most common subtype of lung cancer cases worldwide, originates mainly from the bronchial mucosal epithelium, with a few originating from the mucous glands of the large bronchi, and is characterized by highly infiltrative and destructive growth. The clinical manifestations of LUAD patients are not typical and specific, so some patients have reached the middle and advanced stages of the disease when they are diagnosed, resulting in a poor prognosis, and the 5-year survival rate is only 5% (3–5). Therefore, it is important to study the factors affecting the progression of LUAD and to develop reliable indicators for clinical prognosis prediction.

In recent years, increasing researchers have shifted their attention to the interaction between tumors and immune cells in the tumor immune microenvironment to achieve breakthroughs in tumor therapy (6, 7). The immune microenvironment affects the survival, proliferation, and migration of tumor cells in terms of cytokine secretion and immune cell recruitment (8). Among them, infiltrating M2 macrophages play a particularly important role. M2 macrophages evolve from macrophages in an extremely complex tumor microenvironment and play an important role in controlling tumor growth, invasion, and metastasis. Numerous studies have shown that patients with a large infiltration of M2 macrophages within the tumor tissue or around the cancer tend to have a poor prognosis (9–12). However, a comprehensive analysis of the biological role of M2 macrophages in LUAD tumor progression and clinical prognosis is still lacking today. Therefore, it is important to comprehensively assess the association between M2 macrophages and tumor progression and clinical drug therapy, to develop risk profiles based on M2 macrophages that can accurately predict the prognosis of LUAD patients, and to generate individualized therapy to improve outcomes.

Tumor mutational burden (TMB), defined as the total number of somatic genetic coding errors, base substitutions, insertions, or deletions detected per million bases, has been recognized as a key indicator of benefit from immune checkpoint inhibitors (ICIs) in LUAD patients and is an independent and effective prognostic predictor for LUAD patients (13–17). Theoretically, the more somatic mutations in tumor cells, the higher the TMB value will be, and the greater the likelihood of neoantigen formation and recruitment of more immune cells in and around the tumor immune infiltration microenvironment (TIME). Therefore, TMB can influence TIME (18). Thus, it was possible to use TMB to respond to the clinical treatment effect of ICI (13). It has been reported that the combination of TMB levels and immune infiltration can predict immunotherapy outcomes and clinical prognosis in patients with LUAD (19–21). It is of great significance to further explore the biological role of TMB.

In our study, TCGA-LUAD (The Cancer Genome Atlas-lung adenocarcinoma) database was used to investigate the potential role of M2 macrophage-related genes in LUAD tumor progression and clinical prognosis, and data extracted from the GEO (Gene Expression Omnibus) database were used for external validation. We used the CIBERSORT algorithm to find the most LUAD-related gene modules among M2 macrophage-related genes and developed a weighted gene co-expression network analysis (WGCNA). After multiple methods of hierarchical screening, two genes significantly associated with LUAD were finally selected. The risk score (RS) based on the clinical prognostic contribution of these two genes was calculated for each sample, and all samples were divided into two groups based on the median RS. Then, we developed and validated prognostic line plots based on risk signatures and other clinical variables. Finally, we explored the interrelationship between risk signature and TMB and TIME, investigated the differences in signaling pathways between different RS subgroups, and analyzed the impact of risk signature on the treatment effects of immunotherapy and chemotherapy. In summary, we established an RS based on M2 macrophage-related genes for therapeutic management and clinical prognosis prediction of LUAD patients.

## Materials and Methods

### Data Download and Preprocessing

Our transcriptome data include both TCGA-LUAD and GSE68571 cohorts. The TCGA-LUAD cohort consisted of 595 RNA sequencing samples, including 59 normal samples and 535 tumor samples. We removed samples without clinical follow-up information from TCGA-LUAD cohort, resulting in 504 tumor samples. We also downloaded somatic mutation data from TCGA database for further analysis of copy number variation (CNV). We obtained the GSE68571 cohort from the GEO database as an external validation dataset. Microarray data of GSE68571 were obtained from Affymetrix Human Full Length HuGeneFL Array, and the normalized matrix file was downloaded directly. All cases in the GSE68571 cohort contain survival information. The Human Protein Atlas (http://www.proteinatlas.org) was used to investigate the protein levels of genes.

### Landscape of Infiltrating Immune Cells

CIBERSORT is a tool for deconvolution of expression matrices of human immune cell subtypes based on the principle of linear support vector regression (22–24). We used the CIBERSORT algorithm to analyze the microarray expression matrix of TCGA-LUAD patients to obtain the abundance of 22 tumor-infiltrating immune cell (TIC) subtypes.

### Weighted Gene Co-Expression Network Analysis

The purpose of WGCNA was to find co-expressed gene modules and explore the association between gene networks and phenotypes of interest, as well as core genes in the network (25–27). We used the expression of 16,816 genes from TCGA-LUAD cohort as data and the CIBERSORT results as phenotypes of concern. The soft threshold power (β) from 1 to 20 was selected as a candidate, and the corresponding power values were calculated using the pick Soft Threshold function, the best power value was selected to build the proximity matrix, and our gene distribution was made to conform to the scale-free network according to the connectivity. Using the TOM matrix obtained from gene expression, the genes were again continued to be clustered, the minimum number of module genes was set, and the gene clustering results were cut to obtain different gene modules. The “dynamic tree cutting” algorithm was used to introduce similar genes into the same candidate module. The correlation analysis between the module feature genes and the phenotype of interest was performed by Pearson correlation test (p < 0.05). Our study targeted “M2 macrophages,” so the most significantly correlated modules with M2 macrophages were extracted. All the above analysis was done based on WGCNA and limma packages.

### Cox Regression Analysis and Lasso Regression Analysis

To explore the prognostic role of M2 macrophage-related genes, we used 183 genes from the “greenyellow” module for the next step of the screen. The genes associated with patient survival were first obtained using a univariate regression analysis. Next, to prevent overfitting of the model, lasso regression was performed by generating a penalty function to compress the coefficients of the variables. The results of the lasso regression analysis were incorporated into a multivariate Cox regression analysis to finalize the results for the M2 macrophage-related genes considered to affect the prognosis of LUAD patients.

### Validation of the Prognostic M2 Macrophage-Related Signature

TCGA cohort was used as our training set to calculate the risk score (RS) based on the expression of prognosis-related genes and regression analysis coefficient values. the equation is shown below:


$$riskscore=\sum_{i=1}^{n}\left(coefi*Xi\right)$$

We classify the cases into high-risk group (HRG) and low-risk group (LRG) according to the median RS. Kaplan–Meier (KM) curves were plotted, and the difference in survival between the two groups of LUAD patients was assessed using the log-rank method. Besides, time-dependent receiver operating characteristic (ROC) curves were analyzed to validate the prognostic values. For external validation, we compared the differences in clinically relevant variables between the HRG and LRG groups of patients by the “pheatmap” R package.

### Establishment and Verification of the Nomogram

To more accurately predict patient survival at 1, 3, and 5 years, our nomogram incorporates RS and clinically relevant variables. To more accurately predict patient survival at 1, 3, and 5 years, our nomogram incorporates RS and clinically relevant variables based on the survivor, regplot, and rms software packages. Calibration curves were used to demonstrate the validity of the model.

### Gene Set Enrichment Analysis

The c2.cp.kegg.v7.4.symbol and c5.go.v7.4.symbol collection was used to explore the function annotation by Gene Set Enrichment Analysis (GSEA) software. Results with p value <0.05 were considered statistically significant. The first eight results were selected for visualization.

### Correlation Between Tumor Mutation Burden and Risk Score

Data on somatic mutations in TCGA-LUAD cohort were obtained from TCGA database. The “maftools” R package was used to plot waterfall plots for both HRG and LRG groups. In addition, according to the median mutation load and RS of LUAD patients, we plotted survival curves between the four subgroups.

### Correlation of Risk Score With Tumor Immune Microenvironment Characterization

To explore the correlation between RS and TICs, we used seven methods to assess immune cell infiltration in the tumor microenvironment, including XCELL, TIMER, QUANTISEQ, MCPcounter, EPIC, CIBERSORT, and CIBERSORT-ABS. The ESTIMATE algorithm, which can be based on gene expression data, estimates the stromal score and immune score of a tumor sample for representing the presence of stromal and immune cells. The two scores are summed to obtain the ESTIMATE score, which can be used to estimate tumor purity. Correlation between RS and TICs was performed using Spearman correlation analysis.

### Gene Set Variation Analysis

We used the MSigDB database (https://www.gsea-msigdb.org/gsea/msigdb) for pathway analysis (28). To assess relative pathway activity in individual samples, we performed Genome Variation Analysis (GSVA) (29) using the GSVA package to assign pathway activity estimates.

### Immunotherapy Prediction

Immune checkpoints have been defined as key targets for the inhibition of immune cell function. In this study, we analyzed the expression levels of 47 immune checkpoint blockage-related genes in HRG and LRG. Immunophenoscore (IPS) determines the immunogenicity of a tumor and predicts the response to immune checkpoint inhibitor therapy. IPS calculates scores for each of the four different immunophenotypes (antigen-presenting, effector, suppressor, and checkpoint), and the IPS z-score is an integration of all four, and the higher the IPS z-score, the more immunogenic the sample.

### Prediction of Chemotherapeutic Effect

To investigate the drug sensitivity differences between HRG and LRG, we constructed a ridge regression model based on the Genomics of Drug Sensitivity in Cancer (GDSC) cell lines and TCGA gene expression profiles. Using the pRRophetic algorithm, the half-maximal inhibitory concentrations (IC50) of four chemotherapeutic agents (metformin, cisplatin, paclitaxel, and gemcitabine) were estimated in LUAD patients.

### Statistical Analysis

The Wilcoxon test was used to compare two groups, whereas the Kruskal–Wallis test was used to compare more than two groups. Survival analysis was performed by the Kaplan–Meier log-rank test. The chi-square test was used for analysis between RS and TMB, and Spearman analysis was used to calculate the correlation between the coefficients. A two-sided p < 0.05 was considered statistically significant. All statistical calculations were done in R software (version 4.1.1).

## Results

### Landscape of TIME in LUAD

**Figure 1:**
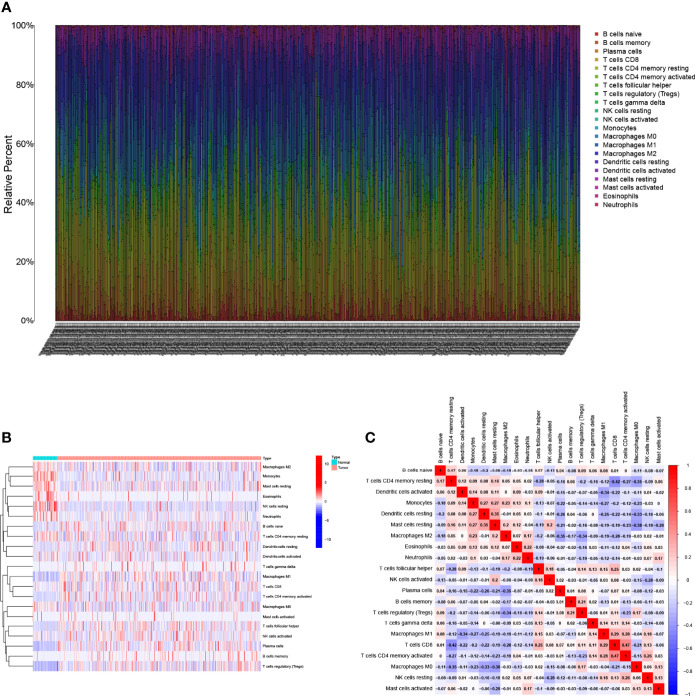
Landscape of immune cell infiltration in the tumor immune environment of LUAD. Subpopulation of 22 immune cell subtypes **(A)** and proportional heatmap of 22 TICs in each LUAD samples **(B)**. **(C)** Intrinsic correlation of 22 infiltrating immune cells in LUAD.

The characteristics of the cases enrolled in this study after preprocessing are shown in **Table 1**. In TCGA-LUAD cohort, complete follow-up information was available for 504 samples. Survival data of the patients showed that 36.31% of the patient endpoint events were death. The median follow-up for the two cohorts was 1.34 years. In the GSE68571 cohort, complete follow-up information was available for 86 samples. Survival data of the patients showed that 27.91% of the patient endpoint events were death. The median follow-up for the two cohorts was 2.42 years. The abundance of 22 TIC subtypes in TCGA-LUAD patients was obtained using the CIBERSORT algorithm (**Additional File 1: Table S1**), as shown in **Figure 1A**. Each column represents a sample, and different colors represent the corresponding proportion of TICs in each sample. We used the proportion of various immune cells in each sample to represent the TIME in the sample to reveal the landscape of TIME in LUAD. However, a comprehensive heatmap based on TIME patterns and clinical phenotypes (**Figure 1B**) visually demonstrated differences in immune cell infiltration between normal and immune tissues. **Figure 1C** illustrates the potential connections among the 22 TICs for a better understanding of TIME.

**Table 1:** Clinicopathological characteristics of LUAD patients from TCGA and GSE68571 databases.

Characteristics	TCGA-LUAD cohort N=504	GSE68571 N=86
**Age**		
<=65	238 (47.22%)	50 (58.14%)
>65	256 (50.79%)	36 (41.86%)
Unknow	10 (1.99%)	0 (0.00%)
**Gender**		
Female	270 (53.57%)	51 (59.30%)
Male	234 (46.43%)	35 (40.70%)
**Stage**		
I-II	389 (77.17%)	NA
III-IV	107 (21.24%)	NA
Unknow	8 (1.59%)	NA
**T**		
T0-T2	437 (86.70%)	NA
T3-T4	64 (12.70%)	NA
Unknow	3 (0.60%)	NA
**M**		
M0	335 (66.47%)	NA
M1	25 (4.96%)	NA
Unknow	144 (28.57%)	NA
**N**		
N0-N1	419 (83.13%)	NA
N2-N3	73 (14.49%)	NA
Unknow	12 (2.38%)	NA
**Survival status**		
Alive	321 (63.69%)	62 (72.09%)
Dead	183 (36.31%)	24 (27.91%)
**The median follow-up time** **(year)**	1.84	2.42

### Establishment of the WGCNA Network

**Figure 2:**
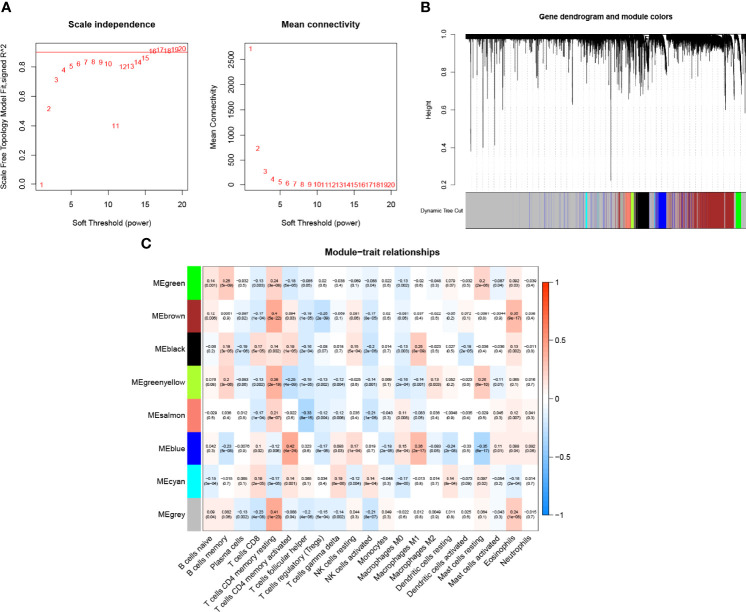
Choosing an appropriate soft threshold (power) and building a hierarchical clustering tree. **(A)**The choice of the soft threshold enables the scale-free topology to achieve an exponent of 0.90, and the average connectivity for 1–20 soft threshold powers is analyzed. **(B)** M2 macrophage-related genes with similar expression patterns were merged into the same module using a dynamic tree-cutting algorithm, creating a hierarchical clustering tree. Heatmap of correlations between **(C)** modules and immune-infiltrating cells (traits).

We developed the WGCNA co-expression network using a sequencing file containing 16,816 genes as well as immune infiltration subpopulations. The scale-free network was constructed by setting the optimal soft threshold power (β = 15) to the first set of power values when the scale-free topology index reached 0.9 (**Figure 2A**). Genes with the same or similar expression patterns were grouped into the same gene module using a “dynamic tree cutting” algorithm (module size = 60) to form a hierarchical clustering tree. Weighted hierarchical clustering analysis was performed, and then its results were segmented, resulting in eight gene modules (**Figure 2B**). The Pearson correlation of each TIC with each candidate module is shown in **Figure 2C**. It was easily observed that the “greenyellow” module (**Additional File 1: Table S2**) had the strongest correlation with M2 macrophages (r = 0.13, p = 0.003). The full complementary procedure on WGCNA is presented in **Figures S1A–D**.

### Development of Risk Signatures

**Figure 3:**
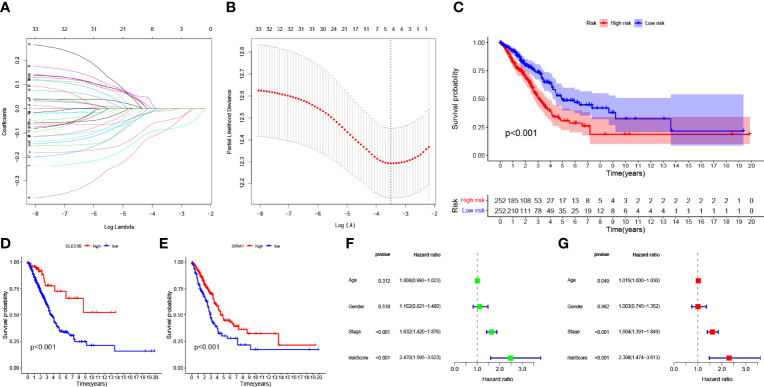
**(A)** Variation curve of the regression coefficient with Log (λ) in Lasso regression. **(B)** Ten-fold cross-validation for tuning parameter selection in lasso regression. Vertical lines are drawn from the best data according to the minimum criterion and 1 standard error criterion. **(C)** Kaplan–Meier curve analysis showing the difference in overall survival between high-risk and low-risk groups in TCGA-LUAD cohort. Kaplan–Meier curve analysis showed the difference in overall survival of the *CLEC3B* **(D)** gene and *GRIA1* **(E)** gene between the high expression group and low expression group. **(F)** Univariate Cox regression results for overall survival. **(G)** Multivariate Cox regression results for overall survival.

Expression data and follow-up information were extracted from TCGA-LUAD project to analyze the impact of M2 macrophage-related genes on the prognosis of LUAD patients. Univariate Cox regression analysis was performed on 183 genes in the candidate module “greenyellow,” and 33 genes were screened (p < 0.05, **Additional File 1: Table S3**). To prevent overfitting, we performed lasso regression analysis on the screened genes and determined the optimal value of the penalty parameter by cross-validation (**Figures 3A, B**). Cox regression analysis was performed on the genes screened by lasso regression analysis, and two M2 macrophage-related genes (*GRIA1* and *CLEC3B*, all HR <1, **Table S4**) that were beneficial to predict the prognosis of LUAD patients were finally identified. The HPA database was used to explore protein expression levels in LUAD samples. We extracted IHC from the HPA database for four cases. Two of them were normal lung tissues, and two were lung cancer tissues. Of the four patients, only one was older than 65 years. All cases were women. The results showed the difference in protein expression of the hub genes (*GRIA1* and *CLEC3B*) in normal and lung cancer tissues (**Additional File: Figures S2A–D**).

Subsequently, two hub genes were incorporated into the risk profile of LUAD patients. The RS was computed:


risk score (RS)=−(0.2400×GRIA1)−(0.1158×CLEC3B)

Finally, the LUAD samples were divided into HRG and LRG based on the median value of RS.

### Validation of Risk Prognostic Features

The K–M survival curve indicated that LRG had better survival outcomes compared to HRG (**Figure 3C**). According to the median expression of the *CLEC3B* gene in the samples, the samples were divided into a high CLEC3B gene expression group and a low *CLEC3B* gene expression group. The K–M survival curve (**Figure 3D**) showed that the *CLEC3B* gene had a significant effect on the prognosis of LUAD patients, and the screening process for M2 macrophage-related genes was very reliable. As with the *CLEC3B* gene, we also performed the same validation for the *GRIA1* gene (**Figure 3E**). Through univariate cox regression analysis and multivariate Cox regression analysis, we obtained hazard ratios (HR) for the risk signature to be 2.470 (95% CI 1.595−3.823; **Figure 3F**) and 2.308 (95% CI 1.474−3.613; **Figure 3G**). These results all consistently indicate that the M2 macrophage-related genes *GRIA1* and *CLEC3B* have good predictive power for clinical outcomes, and the risk signature is an independent prognostic indicator for LUAD.

**Figure 4:**
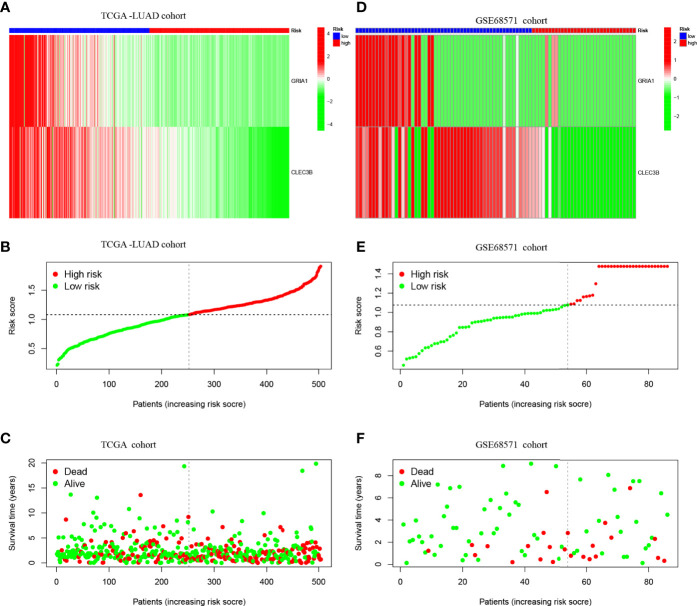
**(A)** Confirmation of prognostic risk scores in the TCGA cohort. **(B)** Polygenic model risk score distribution in TCGA cohort. **(C)** Survival status and duration of LUAD patients in TCGA cohort. **(D)** Confirmation of prognostic risk scores in the GSE68571 cohort. **(E)** Polygenic model risk score distribution in the GSE68571 cohort. **(F)** Survival status and duration of LUAD patients in the GSE68571 cohort.

**Figures 4A–C** shows the expression patterns of the two genes in TCGA-LUAD cohort, the distribution of sample survival status, and corresponding risk scores. Both internal validation with the TCGA-LUAD cohort and external validation with the GSE68571 cohort (**Figures 4D–F**) demonstrated a stable and robust prognostic value for this risk prognostic feature.

### Functional Analysis of M2 Macrophage-Related Genes

**Figure 5:**
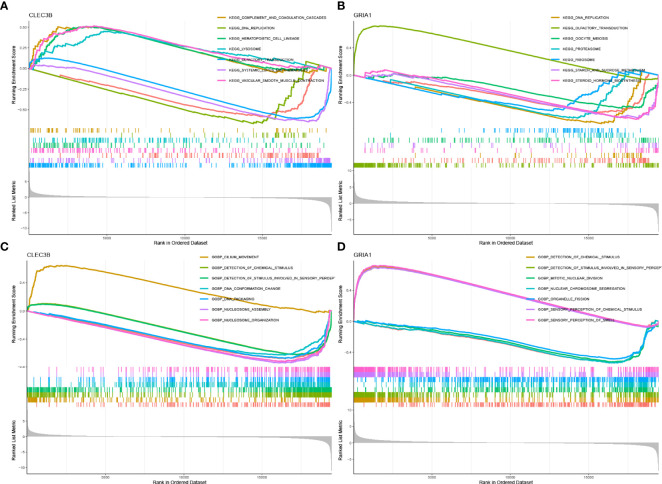
GSEA of samples with high and low expressions of two hub genes. **(A)** Gene set of samples enriched in *CLEC3B* expression collected in KEGG. **(B)** Gene set of samples enriched in *GRIA1* expression collected in KEGG. **(C)** Gene set of samples enriched in *CLEC3B* expression collected at GOBP. **(D)** Gene set of samples enriched in *GRIA1* expression collected at GOBP.

According to the median expression of the *CLEC3B* gene in the samples, the samples were divided into *CLEC3B* gene high expression group and *CLEC3B* gene low expression group. Then, GSEA was performed to identify the functional enrichment of high and low *CLEC3B* gene expression. KEGG enrichment items indicated that high *CLEC3B* gene expression was related to complement and coagulation cascades, vascular smooth muscle contraction, hematopoietic cell lineage, and lysosome signaling pathways (**Figure 5A**). GOBP enrichment items indicated that the high expression of the *CLEC3B* gene was related to the cilium movement signaling pathway (**Figure 5C**).

Similarly, we performed the same functional enrichment process for the *GRIA1* gene as the *CLEC3B* gene. The KEGG enrichment term indicated that the high expression of the *GRIA1* gene was related to the olfactory transduction signaling pathway (**Figure 5B**). The GOBP enrichment term indicated that the high expression of the *GRIA1* gene was related to the sensory perception of chemical stimulus and the sensory perception of the smell signaling pathway (**Figure 5D**).

### Correlation of Risk Characteristics With Clinicopathological Variables

**Figure 6:**
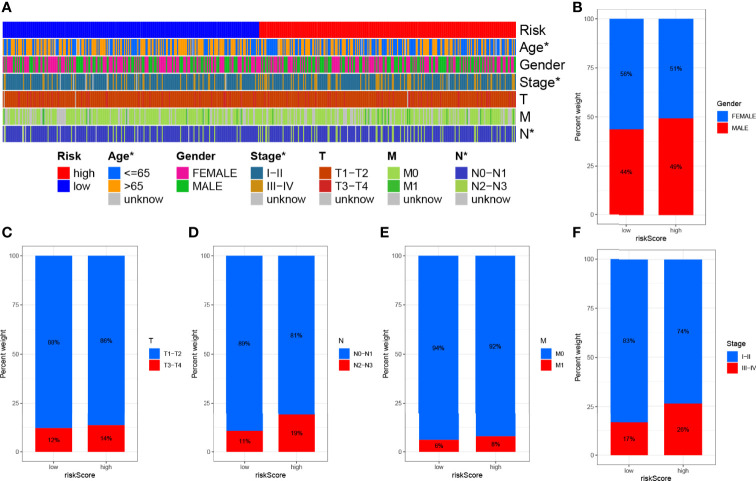
Clinical significance of prognostic risk characteristics. **(A)** Heatmap showing the distribution of clinical characteristics and corresponding risk scores in each sample. Incidence of clinical variable subtypes of LRG/HRG. **(B)** Gender, **(C)** stage T, **(D)** stage N, **(E)** stage M, and **(F)** clinical stage.

Subsequently, to visualize the distribution of clinical variables in the LRG/HRG subgroup, we plotted **Figure 6A**. Clinical subtype scores between HRG and LRG based on gender, stage T, stage N, stage M, and clinical stage are shown in **Figures 6B–F**.

### Construction of Prognostic Nomogram

**Figure 7:**
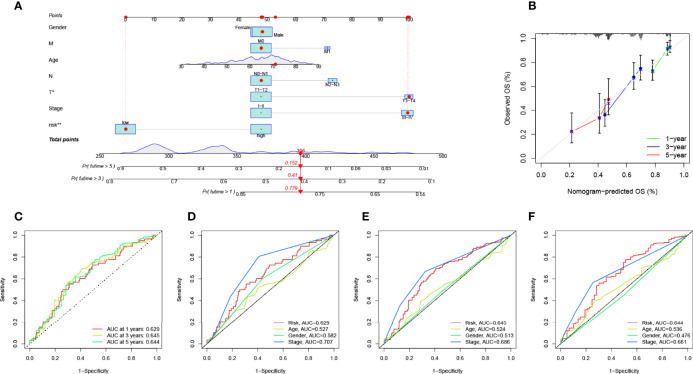
Validation of prognostic efficiency of risk signatures. **(A)** The nomogram was used to predict survival in LUAD patients. **(B)** One-, 3-, and 5-year nomogram calibration curves. **(C)** ROC analysis was used to estimate the predictive value of prognostic features. The area under the **(D–F)** curve (AUC) of the risk score for predicting overall survival at 1, 3, and 5 years and other clinical characteristics.

We plotted ROC curves to estimate the predictive value of prognostic features. The AUC values for 1-, 3-, and 5-year overall survival (OS) reached 0.629, 0.645, and 0.644, respectively, indicating high prognostic validity (**Figure 7C**). Combining risk signature, gender, age, and clinical stage, we performed AUC analysis for 1-year (**Figure 7D**), 3-year (**Figure 7E**), and 5-year OS (**Figure 7F**) and found that risk signatures outperformed across multiple clinicopathological variables. Finally, to more intuitively quantify the effects of risk signature, gender, age, T, N, and M stage, and clinical stage on OS in patients with LUAD, we drew a prognostic nomogram (**Figure 7A**). Nomogram can quantify the clinical characteristics of a patient to be able to visually predict the probability of survival of an individual. For example, in a 71-year-old female LUAD patient with T3N1M0, stage III, low RS, a total score of 396 can be calculated from the nomogram, with survival rates of 77.9%, 41%, and 15.2% at 1, 3, and 5 years, respectively. We used a calibration curve for validation, and the results demonstrated that the nomogram has good prognostic performance (**Figure 7B**).

### Association of Risk Signature With TMB

**Figure 8:**
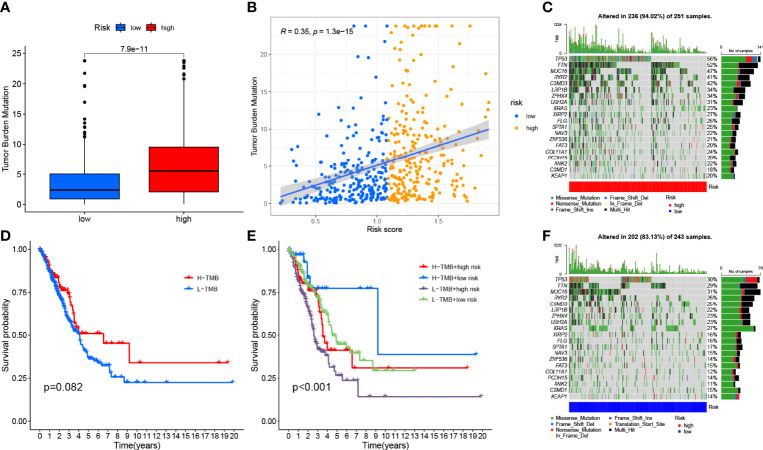
Correlation between risk score and TMB. **(A)** Differences in TMB between HRG and LRG. **(B)** Scatterplots depicting the positive correlation between risk scores and TMB. **(D)** Kaplan–Meier curves of high TMB and low TMB groups. **(E)** Kaplan–Meier curve stratification of patients according to TMB and risk signature. The oncoPrint was constructed using high risk score **(C)** and low risk score **(F)**.

To explore the potential link between TMB and risk signatures, we compared the TMB of LRG and HRG samples and found that HRG had higher TMB (p < 0.001, **Figure 8A**). The RS and TMB of each sample are shown in **Figure 8B**. To analyze the effect of TMB on OS in LUAD patients, we divided the total sample into high-TMB and low-TMB groups according to the median TMB and plotted a K–M survival curve (p = 0.082, **Figure 8D**). **Figure 8E** can visually demonstrate the synergistic effect of RS and TMB in the prognosis of LUAD patients.

In addition, we investigated the type and distribution of somatic gene mutations in different RS subgroups, mapping a comprehensive landscape of HRG and LRG somatic variation (**Figures 8C, F**). Significantly mutated gene (SMG) mutation profiles indicated that *TP53* (56% vs. 30%), *TTN* (52% vs. 29%), and *MUC*16 (47% vs. 31%) experienced higher somatic mutations in HRG core subtype rate, while *FGFR3* (27% vs. 23%) had a higher rate of somatic mutation in LRG.

### Risk Signature in TIME Context

**Figure 9:**
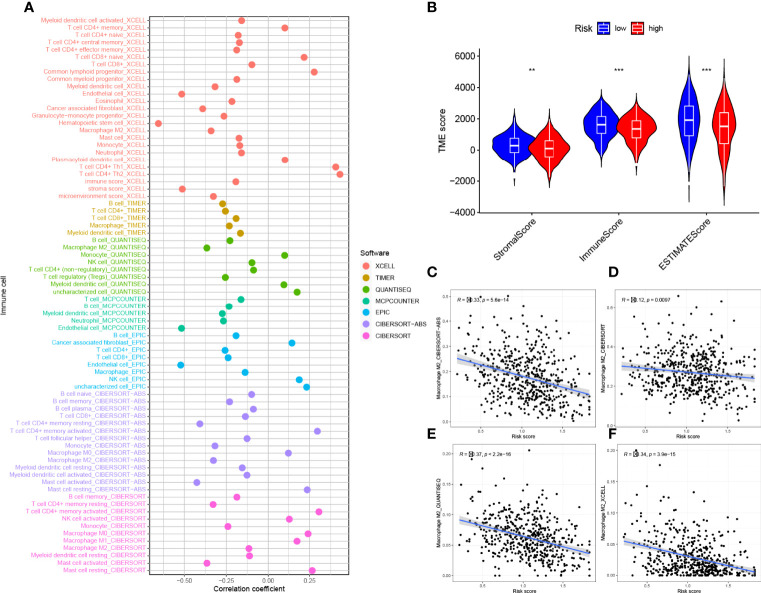
Estimated abundance of tumor-infiltrating cells. Patients in the **(A)** high-risk group had a stronger correlation with tumor-infiltrating immune cells, as shown by the Spearman correlation analysis. **(B)** Association between prognostic risk signatures and central immune checkpoint genes. The asterisks represented the statistical p value (**P < 0.01; ***P < 0.001).The correlations predicted by the four methods CIBERSORT−ABS **(C)**, CIBERSORT **(D)**, QUANTISEQ **(E)**, and XCELL **(F)** were validated.

We investigated the potential association between risk signatures based on M2 macrophage-related genes and TIME, using Spearman correlation to analyze the two, and plotted them for easy observation (**Figure 9A**, **Table S5**). The results of ESTIMATE analysis showed that immune score, stromal score, and ESTIMATE scores in HRG tended to decrease significantly (p < 0.01, **Figure 9B**). Validation of the correlations predicted by the four methods CIBERSORT−ABS (**Figure 9C**), CIBERSORT (**Figure 9D**), QUANTISEQ (**Figure 9E**), and XCELL (**Figure 9F**) showed that our analysis was accurate.

### Enrichment of Signaling Pathways in Low-/High-Risk Groups

**Figure 10:**
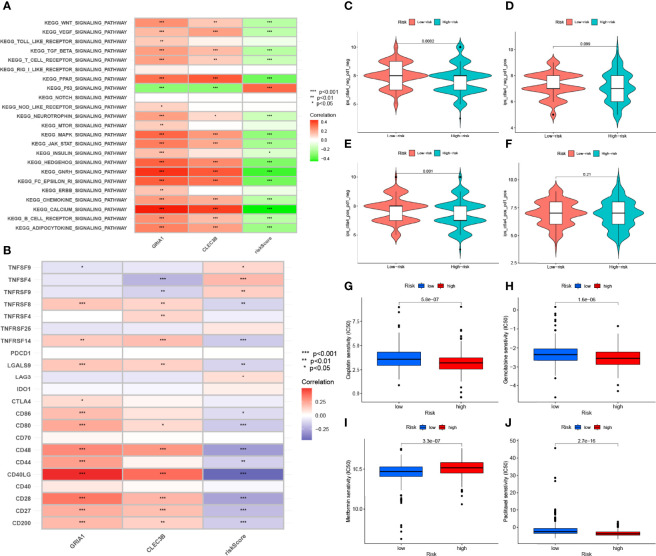
Enrichment pathways for GSVA. **(A)** heatmap showing the correlation of representative pathway items with KEGG with risk scores. Predicting immunotherapy response. **(B)** Association of immune checkpoint blockade gene expression levels with risk scores. **(C–F)** IPS score distribution map. Estimates of chemotherapy effect risk scores. Sensitivity analysis of **(G)** cisplatin in patients with high and low risk scores. Sensitivity analysis of **(H)** gemcitabine in patients with high and low risk scores. **(I)** Sensitivity analysis of metformin in patients with high and low risk scores. **(J)** Sensitivity analysis of paclitaxel in patients with high and low risk scores.

By GSVA analysis (**Figure 10A**), we could easily find that the expression levels of the *CLEC3B* gene and *GRIA1* gene were negatively correlated with the p53 signaling pathway, while the calcium signaling pathway, KEGG/PPAR signaling pathway, KEGG/GNRH signaling pathway, and FC epsilon RI signaling pathways such as signaling pathway are positively correlated; RS is positively correlated with the p53 signaling pathway and negatively correlated with the calcium signaling pathway, KEGG/PPAR signaling pathway, KEGG/GNRH signaling pathway, FC epsilon RI signaling pathway, and other signaling pathways.

### Immunotherapy Prediction

Since there is no data information of immunotherapy in TCGA-LUAD dataset, we used the expression levels of genes related to immune checkpoint blockade to represent the effect of immunotherapy. Our study found that most immune checkpoint blockade-related genes (i.e., *CD40LG*, *CD48*, *TNFRSF14*, *CD80*, *CD200*, *TNFRSF8*) were significantly negatively correlated with risk signatures, and a small number of immune checkpoint blockade-related genes (such as *TNFSF4* and *TNFRSF9*) were positively correlated with the risk signature (**Figure 10B**). HRG had higher IPS scores in this risk scoring system (PD1-negative and CTLA4-negative, **Figure 10C**). It indicates that high-risk patients are more suitable for novel ICI immunotherapy. LRG was more suitable for CTLA4 immunosuppressive therapy alone (PD1-negative and CTLA4-positive, **Figure 10E**). These results all confirm a potential association between risk scores and immunotherapy efficacy (**Figures 10C–F**).

### Prediction of Response to Chemotherapy

Through analysis, we found that the IC50 of the four chemotherapeutic drugs (metformin, cisplatin, paclitaxel, and gemcitabine) showed significant differences in HRG/LRG. The drug sensitivities of cisplatin (**Figure 10G**) and paclitaxel (**Figure 10J**) were higher in HRG than in LRG, whereas gemcitabine (**Figure 10H**) and metformin (**Figure 10I**) had higher drug sensitivities in LRG. These results suggest a potential link between the risk signature and chemotherapeutic drug sensitivity.

## Discussion

Lung cancer is a malignant tumor of the respiratory system with a high incidence, and it has the highest mortality rate among both men and women (2). Of these, non-small cell lung cancer (NSCLC) accounts for 80% of all lung cancer pathology types, and half of NSCLC is LUAD. Currently, clinically, the main treatments for LUAD are surgery, systemic chemotherapy, immunotherapy, and targeted therapy. Although these treatment options can significantly change the prognosis of patients with LUAD, the treatment effect is still poor for patients with advanced disease. In recent years, the study of the immune-related tumor microenvironment (TME) has received increasing attention. Moreover, as an important component of TME, M2 macrophages play an important role in antitumor and are promising to be the next target of immunotherapy (30, 31).

Macrophages are an important cellular component of the innate immune system and were once thought to be an important type of cells in the process of antitumor immune regulation. They eliminate tumors by directly killing or presenting tumor-associated antigens to induce immune responses. However, the phenotype of macrophages is very heterogeneous, and at the same time, they can act as negative regulators of the immune system. Under the induction of tumor cells, macrophages can promote the proliferation of tumor cells and inhibit the antitumor activity of T cells and natural killer cells. These cells are called tumor-associated macrophages and express M2-type molecular markers (32). Current studies have found that tumor-associated macrophages are abundantly expressed in LUAD and indicate poor prognosis (33). However, the specific biological role of M2 macrophages in LUAD tumors remains obscure.

In this study, we extracted two cohorts, TCGA-LUAD and GSE68571, from the database, the former for model development and the latter for external validation. Five hundred four tumor samples and 16,816 genes were used to further investigate the potential role of M2 macrophages in LUAD tumor progression and clinical prognosis. The abundance of 22 TIC isoforms was obtained using the CIBERSORT algorithm. WGCNA was used to find gene modules (greenyellow) associated with M2 macrophages. There are 183 genes in this gene module. To verify the favorable prognostic value of these genes for LUAD patients, we combined these genes with clinical information in the samples and finally determined that *GRIA1* and *CLEC3B* genes were significantly associated with prognosis through univariate, LASSO, and multivariate Cox analyses. We used the Cox regression HR of each gene as the coefficient, calculated the RS in each sample according to the gene expression in the sample, and divided all samples into HRG and LRG according to the median RS to facilitate subsequent research. K–M survival curves and ROC curves indicated that the risk model performed well, which was further confirmed in an external dataset (GSE68571 cohort). These results all indicate that the risk model based on the *GRIA1* gene and *CLEC3B* gene can be used as an independent indicator for predicting the clinical prognosis of LUAD patients.

To clearly show the relationship between the risk signature and the clinical prognosis of LUAD, we combined the risk signature with various clinical variables to construct a prognostic nomogram for evaluating the 1-, 3-, and 5-year survival probability of LUAD patients and verified using the calibration curve. Nomogram is a graph that represents the functional relationship between multiple independent variables in a plane rectangular coordinate system with a cluster of disjoint line segments. It is based on multifactor regression analysis, where multiple predictors are integrated and then plotted on the same plane using scaled line segments at a certain scale and thus used to express the interrelationships among the variables in the prediction model. According to the degree of contribution of each influencing factor to the outcome variable in the model, each value level of each influencing factor is given a score, and then the individual scores are summed to obtain the total score, and finally the predictive value of the individual outcome event is calculated through the functional transformation relationship between the total score and the probability of the occurrence of the outcome event. The nomogram has the advantages of visualization and quantification.

In addition, we enriched signaling pathways using two methods (KEGG and GOBP) to analyze the connections between *GRIA1* and *CLEC3B* genes and signaling pathways.

Studies have shown an association between immunotherapy response and genetic alternation (34, 35). To explore the impact of risk signature and TMB on the clinical prognosis of LUAD, we extracted somatic mutation data from TCGA database and divided the total sample into high-TMB and low-TMB groups based on the median TMB. The association between risk signature and TMB was analyzed, and the two were combined in pairs to compare the differences in survival outcomes between the groups. We found that TMB is an independent predictor of risk signature and has important implications in tumor progression and predicting clinical prognosis.

Currently, cisplatin-based chemotherapy is the basic regimen for LUAD chemotherapy and can significantly improve the median survival time (36, 37). In our study, the drug sensitivity of HRG to cisplatin was significantly higher than that of LRG, while gemcitabine and metformin were more suitable for LRG. For this reason, taking the RS into account when administering chemotherapy drugs to patients with LUAD may lead to better outcomes.

Among the M2 macrophage-related genes we finally screened, the biological function of the *GRIA1* gene in LUAD tumor progression has not been revealed (38). In the past, researchers have paid more attention to the association between the *GRIA1* gene and migraine (39–41), but recently they have begun to turn their attention to the role of the *GRIA1* gene in tumor biology. Some scholars have found that the *GRIA1* gene affects the prognosis of children with acute lymphoblastic leukemia (42). Compared with the *GRIA1* gene, the biological role of the *CLEC3B* gene in tumor is more prominent. The protein encoded by the *CLEC3B* gene is a tetraspanin that can bind to the plasminogen kringle-4 and is mainly located in the extracellular matrix and cytoplasm (43, 44). Tetraspanin can induce the activation of plasminogen to hydrolyze proteins extracellularly, and plasminogen is involved in tumor metastasis and invasion (43, 45–48). Zhu and his colleagues found that the *CLEC3B* gene affects colon cancer tumor progression and is a potential therapeutic factor for colon cancer (44). Dai and his team found that the downregulation of exosomes *CLEC3B* in hepatocellular carcinoma promotes metastasis and angiogenesis through AMPK and VEGF signaling and is a potential therapeutic target for hepatocellular carcinoma (43). Sun and his partners discovered through research that the *CLEC3B* gene affects TIME and serves as a potential prognostic biological marker for *LUAD* (49). This corroborates with our findings. In our study, the effects of the *GRIA1* gene and *CLEC3B* gene on TIME, TMB, and clinical prognosis were elucidated. The study found that the overexpression of the *GRIA1* gene and *CLEC3B* gene is not conducive to the prognosis of LUAD patients. However, the underlying molecular mechanisms of the *GRIA1* gene and *CLEC3B* gene in LUAD have not been elucidated, and further studies are required.

Of course, our study still needs improvement. First, we are still a long way from clinical translation. The next step will be to collect tissue specimens and validate our results at the cellular, animal, and tissue levels, respectively, to make the results more credible.

## Conclusions

In conclusion, M2 macrophage-based risk scores have a major role in estimating prognostic outcomes, TIME heterogeneity, TMB, and treatment response evaluation. In addition, the potential roles of the *GRIA1* gene and *CLEC3B* gene in LUAD were also explored. Nonetheless, these findings require further experimental and clinical validation in different centers and larger cohorts.

## Data Availability Statement

The raw data supporting the conclusions of this article will be made available by the authors, without undue reservation.

## Author Contributions

CX, LS, ZK, LY, XuL, and DP designed this work. YY, YiL, JG, XL, and NL analyzed the data. JL and LS wrote this manuscript. ZK edited and revised the manuscript. All authors approved this manuscript.

## Funding

This study was funded by National Key R&D Program of China (2021YFA0911600), National Natural Science Foundation of China (81773257, 81972867), the Shenzhen Municipal Government of China (RCYX20200714114701035, JCYJ20180507184642475), the Health Care Commission of Henan Province (201403125, LHGJ20190422), 2020 Capital Health Development Research Special Project (2020-2Z-40713), 2020 Peking University Baidu Fund Grant (2020BD033), and the Department of Education of Henan Province (21A320037).

## Conflict of Interest

The authors declare that the research was conducted in the absence of any commercial or financial relationships that could be construed as a potential conflict of interest.

## Publisher’s Note

All claims expressed in this article are solely those of the authors and do not necessarily represent those of their affiliated organizations, or those of the publisher, the editors and the reviewers. Any product that may be evaluated in this article, or claim that may be made by its manufacturer, is not guaranteed or endorsed by the publisher.

## Supplementary Material

Supplementary Figure 1 Data analysis process by using the WGCNA method. **(A)** Sample clustering to detect outliers. **(B)** Gene clustering on TOM−based dissimilarity. **(C)** Gene dendrogram and module colors. **(D)** Clustering of module eigengenes.

Supplementary Figure 2 Differentially expressed proteins of *CLEC3B* in normal **(A)** and LUAD tissues **(B)** in the Human Protein Atlas database. Differentially expressed proteins of *GRIA1* in normal **(C)** and LUAD tissues **(D)** in the Human Protein Atlas database.

Supplementary Table 1 The Results of CIBERSORT algorithm.

Supplementary Table 2 The genes and corresponding modules after WGCNA.

Supplementary Table 3 The results of univariate regression analysis.

Supplementary Table 4 The results of multivariate regression analysis.

Supplementary Table 5 The results of correlation of risk score with immune infiltrating cells.

## Abbreviations

LUAD, lung adenocarcinoma; WGCNA, weighted gene co-expression network analysis; RS, risk score; HRG, high-risk group; LRG, low-risk group; NCI, National Cancer Institute; TMB, tumor mutational burden; ICIs, immune checkpoint inhibitors; TIME, tumor immune infiltration microenvironment; TCGA-LUAD, the Cancer Genome Atlas-lung adenocarcinoma; GEO, Gene Expression Omnibus; CNV, copy number variation; TICs, tumor-infiltrating immune cell; KM, Kaplan–Meier; ROC, receiver operating characteristic; GSEA, Gene Set Enrichment Analysis; GSVA, Applied Gene Set Variation Analysis; IPS, Immune Phenomenon Score; GDSC, Genomics of Cancer Drug Sensitivity; IC50s, half-maximal inhibitory concentrations; HR, hazard ratios; OS, overall survival; SMG, significantly mutated gene; NSCLC, non-small cell lung cancer; TME, tumor microenvironment.
