# Supplementary figures and images for: Clinical M2 Macrophage-Related Genes Can Serve as a Reliable Predictor of Lung Adenocarcinoma
Source: Front Oncol. 2022 Jul 22;12:919899. doi: 10.3389/fonc.2022.919899 (PMC9352953; doi:10.3389/fonc.2022.919899)

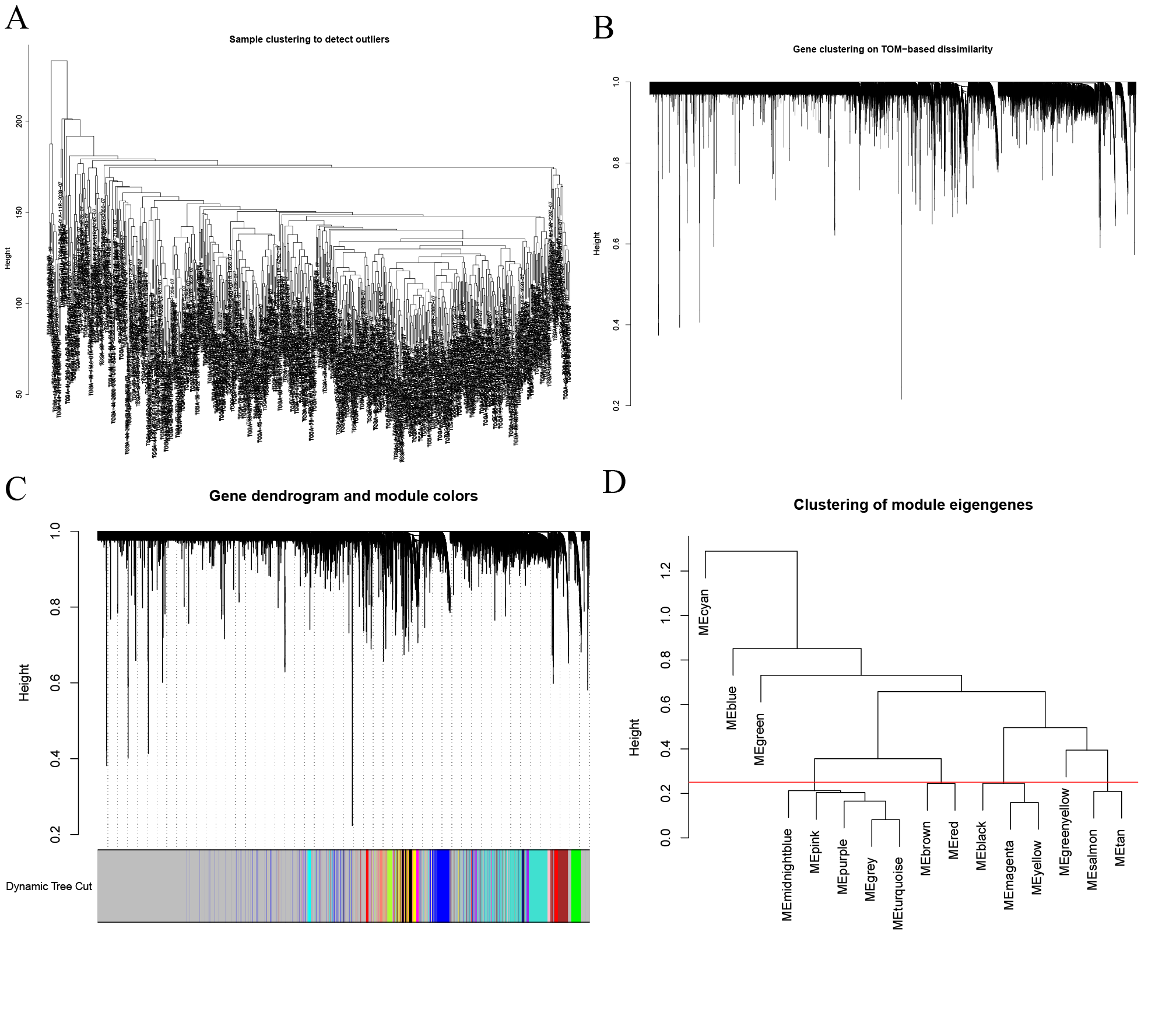

Supplement: Supplementary Figure 1 — Data analysis process by using the WGCNA method. (A) Sample clustering to detect outliers. (B) Gene clustering on TOM−based dissimilarity. (C) Gene dendrogram and module colors. (D) Clustering of module eigengenes. [file Image_1.tif]

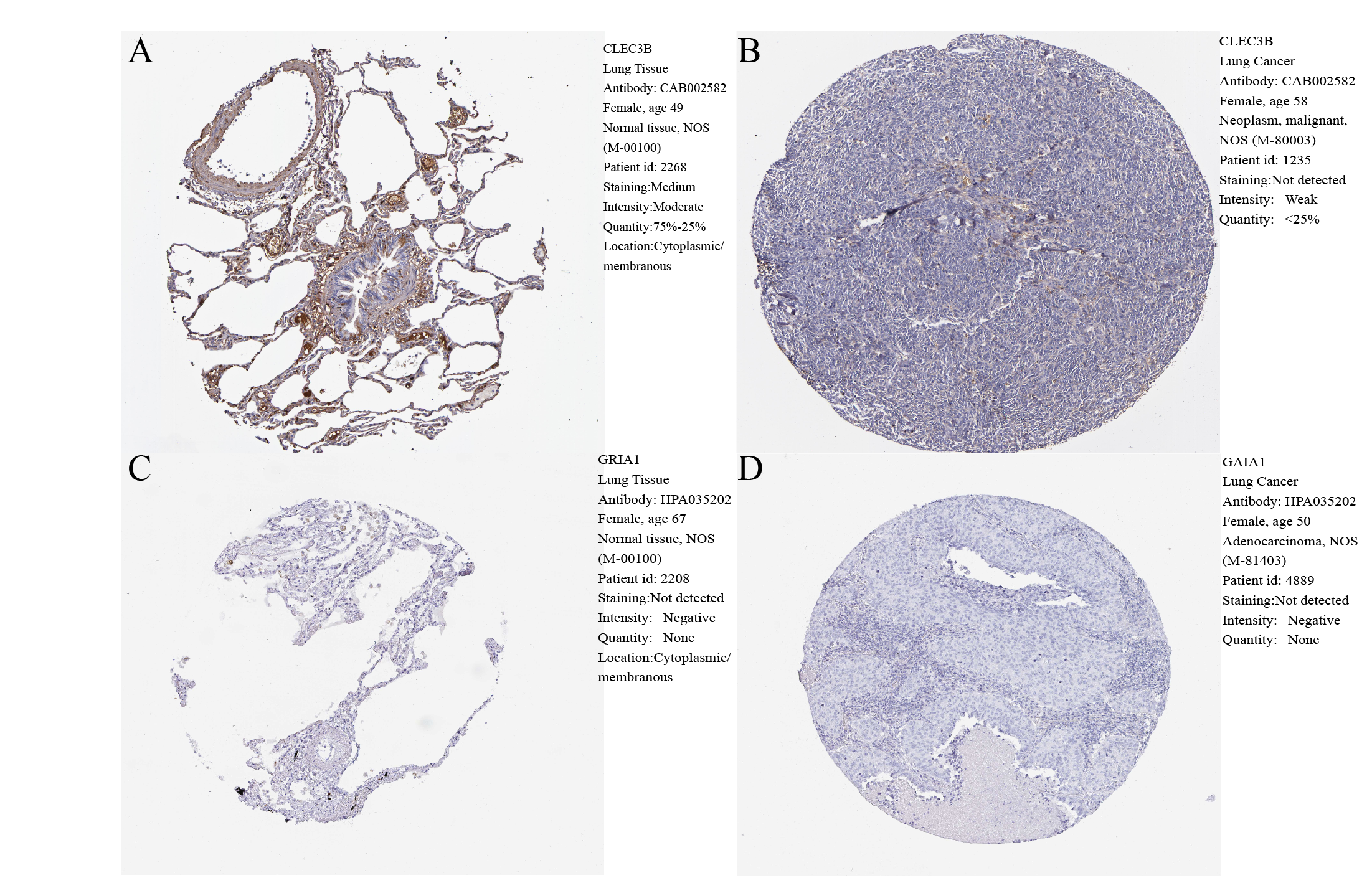

Supplement: Supplementary Figure 2 — Differentially expressed proteins of CLEC3B in normal (A) and LUAD tissues (B) in the Human Protein Atlas database. Differentially expressed proteins of GRIA1 in normal (C) and LUAD tissues (D) in the Human Protein Atlas database. [file Image_2.tif]
